# Effect of the SiCl_4_ Flow Rate on SiBN Deposition Kinetics in SiCl_4_-BCl_3_-NH_3_-H_2_-Ar Environment

**DOI:** 10.3390/ma10060627

**Published:** 2017-06-07

**Authors:** Jianping Li, Hailong Qin, Yongsheng Liu, Fang Ye, Zan Li, Laifei Cheng, Litong Zhang

**Affiliations:** Science and Technology on Thermostructural Composite Materials Laboratory, Northwestern Polytechnical University, Xi’an 710072, Shaanxi, China; ijianping@163.com (J.L.); qinxlong555@163.com (H.Q.); yefang511@nwpu.edu.cn (F.Y.); zli4_07@163.com (Z.L.); chenglf@nwpu.edu.cn (L.C.); zhanglt@nwpu.edu.cn (L.Z.)

**Keywords:** SiBN coating, low pressure chemical vapor deposition, SiCl_4_ flow, deposition mechanism

## Abstract

To improve the thermal and mechanical stability of SiC_f_/SiC or C/SiC composites with SiBN interphase, SiBN coating was deposited by low pressure chemical vapor deposition (LPCVD) using SiCl_4_-BCl_3_-NH_3_-H_2_-Ar gas system. The effect of the SiCl_4_ flow rate on deposition kinetics was investigated. Results show that deposition rate increases at first and then decreases with the increase of the SiCl_4_ flow rate. The surface of the coating is a uniform cauliflower-like structure at the SiCl_4_ flow rate of 10 mL/min and 20 mL/min. The surface is covered with small spherical particles when the flow rate is 30 mL/min. The coatings deposited at various SiCl_4_ flow rates are all X-ray amorphous and contain Si, B, N, and O elements. The main bonding states are B-N, Si-N, and N-O. B element and B-N bonding decrease with the increase of SiCl_4_ flow rate, while Si element and Si-N bonding increase. The main deposition mechanism refers to two parallel reactions of BCl_3_+NH_3_ and SiCl_4_+NH_3_. The deposition process is mainly controlled by the reaction of BCl_3_+NH_3_.

## 1. Introduction

Continuous fiber reinforced silicon carbide ceramic matrix composite (CFCC-SiC) are considered as one of the most promising new generation of thermo-structural materials, which exhibit excellent properties in many aspects, such as high temperature resistance, low density, high specific strength, high specific modulus, antioxidant ablation, and high reliability [1,2]. Interphase is the key microstructure unit for strengthening-toughening of CFCC-SiC and h-BN is widely used as the interphase of SiC/SiC composites [3,4,5]. However, the oxidation resistance of BN interphase is still poor when SiC_f_/SiC or C_f_/SiC composites are used at relatively higher temperatures and in an oxidizing environment. The oxidizing medium could spread to fibers, leading to the oxidation of composites and failure to meet the application requirement under the high temperature oxidizing environment for a long time [4]. Consequently, it is necessary to improve the oxidation resistance of the BN interphase in SiC_f_/BN/SiC and C_f_/SiC composite.

Silicon is one of the high temperature stable elements. What’s more, oxygen diffuses more slowly in SiO_2_ glass than that in B_2_O_3_ glass [6]. In recent study of SiC_f_/BN/SiC composite, BN coating will be oxidized and gasified in 1–10% P_H2O_ environment at 700–800 °C, even though the h-BN was deposited at 1800 °C. The BN coatings deposited at 1060 °C and 1400 °C are even worse. However, the BN interphase containing 22 wt % Si element deposited at 1400 °C survived after work for 1000 h in the same environment [7]. Therefore, doping BN interphase with Si will be an effective way to improve the antioxidant ability of the BN interphase in SiC_f_/BN/SiC composites or C_f_/BN/SiC composites.

Much work has been done in the study of SiBN coating. Moore et al. [8] deposited SiBN interphase at 1400 °C using BCl_3_-NH_3_-SiHCl_3_-H_2_-N_2_-Ar mixture via chemical vapor deposition (CVD). Thanks to the doping of 15–40 wt % Si element, the SiBN interphase enhanced the oxidation resistance of composites 2–3 times more than pure BN at 1200–1500 °C and was more resistant to the reaction with moisture. Nakamura et al. [9] deposited SiBN coating using (C_2_H_5_)_3_SiH (TES)-(C_2_H_5_)_3_B (TDB)-NH_3_ mixture via metalorganic chemical vapor deposition (MOCVD). The coating was amorphous. Results showed that the temperature and the flow ratio of TES and TDB influence the deposition process a lot. Essafti et al. [10] deposited SiBN coating at 800 °C using B_2_H_6_-NH_3_-SiH_4_-H_2_ mixture via CVD. SiBN coating was deposited through two parallel reactions of B_2_H_6_+NH_3_ and SiH_4_+NH_3_ and the flow ratio SiH_4_/B_2_H_6_ determines the micro components of the coating. The amorphous SiBN can be deposited using SiCl_4_-BCl_3_-NH_3_-H_2_-Ar mixture. The effect of temperature on SiBN deposition and the relationship between SiBN interphase and composites’ mechanical properties have been discussed in our previous work [11,12,13,14]. So far, the flow of reactants, an important factor influencing the deposition, has been rarely reported.

In this paper, the SiBN coating was deposited on carbon cloth systematically using SiCl_4_-BCl_3_-NH_3_-H_2_-Ar as the reaction mixture at 900 °C under a SiCl_4_ flow rate ranging from 10 mL/min to 30 mL/min. The effect of SiCl_4_ flow rate on the deposition kinetics—including deposition rate, morphology, phase composition, chemical composition, and bonding states of SiBN coating—was investigated. Additionally, the deposition mechanism was discussed.

## 2. Materials and Methods

### 2.1. Materials and Preparation Method

In the CVD gas system of SiCl_4_-BCl_3_-NH_3_-H_2_-Ar, SiCl_4_ (purity 99.99 wt %) was used as silicon resource; BCl_3_ (purity 99.9 wt %) was used as boron resource; NH_3_ (purity 99.99 wt %) was used as nitrogen resource; H_2_ (purity 99.999 wt %) was the dilution gas and carrier gas which delivered SiCl_4_ from the bubbler to the reactor; and Ar (purity 99.999 wt %) was the protection gas. Carbon cloth woven with T300 carbon fiber (Toray Co., Tokyo, Japan) was used as deposition substrate.

The SiCl_4_ reactant was carried into the reactor by H_2_ via bubbling and the amount of SiCl_4_ delivered by H_2_ could be calculated by the formula

(1) $$F_{T}=\frac{2.24\times 10^{-2}P_{T}F_{C}}{RT}$$

where *F_T_* is the flow rate (mL/min) of SiCl_4_ carried by H_2_; *P_T_* is the saturated vapor pressure (Pa) of SiCl_4_ at certain temperature *T*; *F_C_* is the flow rate (mL/min) of carrier H_2_; *R* is the gas constant (8.314 J/(mol K)); *T* is the temperature (K) of SiCl_4_ liquid. In this work, *T* is set at 30 °C and *P_T_*= 38.738 kPa, according to NIST Chemistry WebBook search. 

Hot-wall CVD reactor was used for SiBN coating deposition, and the deposition parameters are shown in Table 1. Before depositing the SiBN coating, carbon cloths, bunched with carbon fiber, were suspended in the reactor and the reactor was evacuated to the desired pressure and heated up to the deposition temperature. Then the reactants were introduced into the reactor, starting the deposition. After the deposition, reactants and power were cut off. The reactor was filled with argon to the atmospheric pressure when it was cooled down to room temperature. Then samples were removed from the reactor for analysis.

### 2.2. Characterization Method

The surface and cross-section morphologies of T300 carbon cloth after CVD SiBN coating were observed with scanning electron microscopy (SEM, S-2700, Hitachi, Tokyo, Japan). In order to get the deposition rate, 10 different positions distributed on different fibers uniformly around the cross-sectional edge were observed. The deposition rate was calculated via the equations

(2) $$\bar{H}=\frac{H_{1}+H_{2}+\ldots +H_{10}}{10}$$

(3) $$v=\frac{\bar{H}}{t}$$

where the $\bar{H}$ refers to the average thickness (nm) of SiBN coating; *H_i_* (i = 1, 2... 10) refers to the thickness (nm) of SiBN at different positions; *v* refers to the deposition rate (nm/h); *t* refers to the deposition time (h).

X-ray diffraction (XRD, X’Pert pro, Philip; Cu K-Alpha, λ = 0.154 nm) was used to characterize the phase composition of the SiBN coating with a scanning speed of 0.1 s/step with 0.02°/step from 10° to 90°. The element types, element content and chemical bonding states of the SiBN coating were analyzed by X-ray photoelectron spectroscopy (XPS, Al K-Alpha, Thermo Scientific, New York, NY, USA) with pass energy of 100 eV, energy step of 1.0 eV, and number of energy steps is 1361.

## 3. Results and Discussion

### 3.1. Deposition Rate

**Figure 1:**
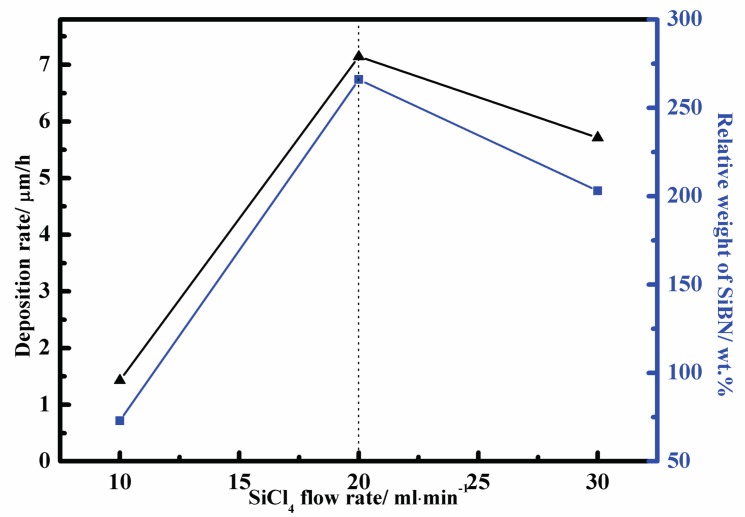
Deposition rate of SiBN coating and average weight percent gain of samples at various SiCl_4_ flow rate.

The deposition rate of SiBN coating and average weight percent gain of samples at various flow rate of SiCl_4_ at 900 °C are drawn in Figure 1. The results indicate that deposition rate varies greatly with the increase of SiCl_4_ flow rate. It increases at first and then decreases. The maximum deposition rate was obtained at about 20 mL/min. After 7 h of deposition the average thickness of SiBN ceramic film was about 50 µm and the average weight percent gain of samples was 266%. The lowest one appears at the flow rate of 10 mL/min with the average deposition thickness of 10 µm and the weight percent gain of 73%. The deposition rate at 30 mL/min is lower than that at 20 mL/min. It does not mean that the reaction rate decreases with the increase of reactant flow rate, but the increase of reaction rate promotes gas nucleation, thus reducing the amount of reactant. This consequently reduces the amount of the material deposited on the substrate. This will be discussed further in Section 3.2.

### 3.2. Morphology

**Figure 2:**
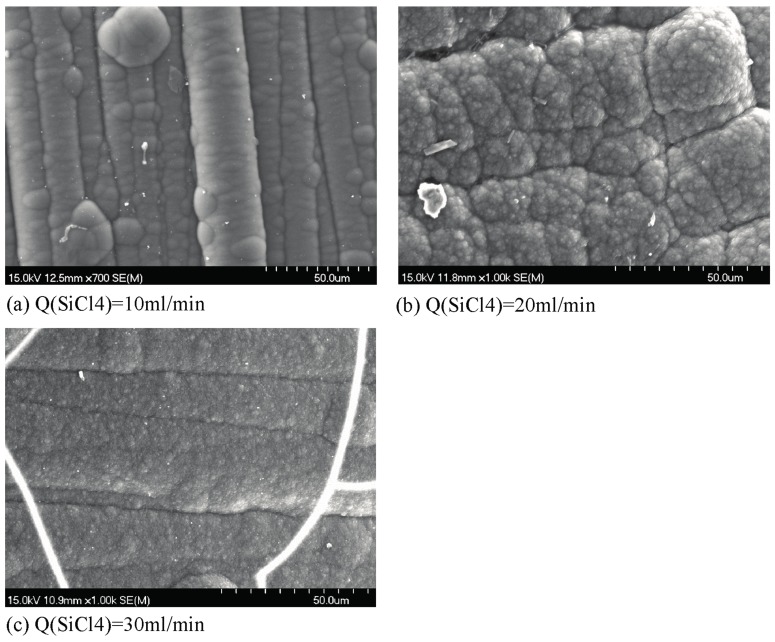
Scanning Electron Microscopy (SEM) images of surface morphologies of SiBN ceramic deposited at various SiCl_4_ flow rates (**a**) Q(SiCl_4_) = 10 mL/min; (**b**) Q(SiCl_4_) = 20 mL/min; (**c**) Q(SiCl_4_) = 30 mL/min.

**Figure 3:**
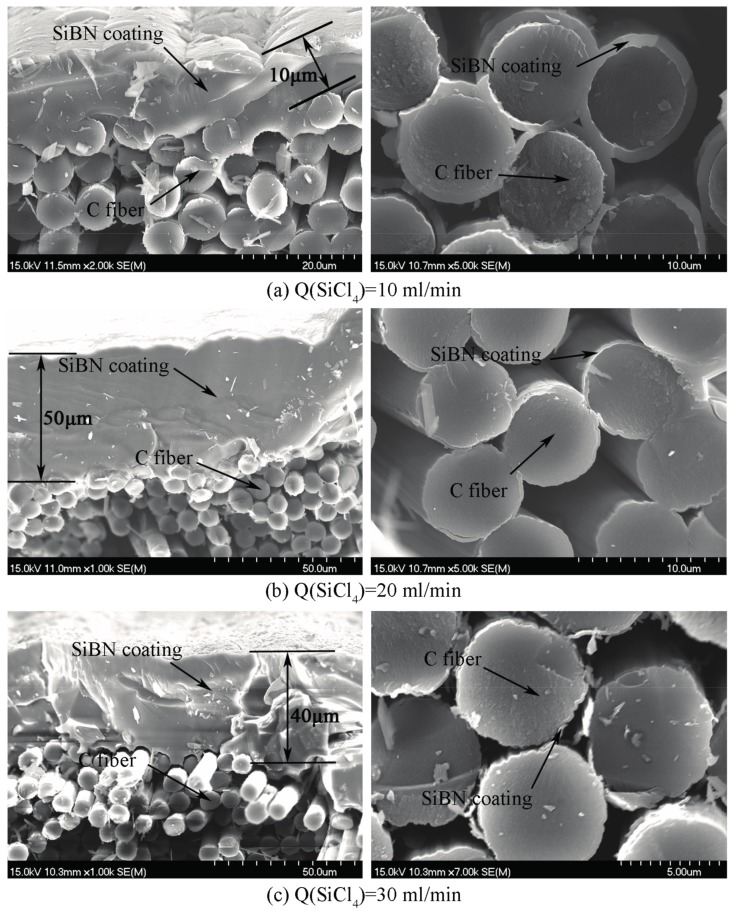
SEM images of cross-section morphologies of SiBN ceramic deposited at various SiCl_4_ flow rates (**a**) Q(SiCl_4_) = 10 mL/min; (**b**) Q(SiCl_4_) = 20 mL/min; (**c**) Q(SiCl_4_) = 30 mL/min.

The SEM images of the surface and cross-section of carbon cloth after CVD SiBN are presented in Figure 2 and Figure 3. As shown in Figure 2, the surface morphologies of SiBN ceramic deposited at various SiCl_4_ flow rates are different from each other. SiBN coating, deposited at the flow rate of 10 mL/min, was a uniform cauliflower-like structure, dense and smooth. When SiCl_4_ flow rate is 20 mL/min, the cauliflower-like structure is bigger than that deposited at 10 mL/min. Besides, there are many small spherical particles closely packed on the surface, which make the surface rough. As the SiCl_4_ flow rate rises to 30 mL/min, the SiBN cauliflower-like structure reduces and smaller spherical particles appear on the rough surface.

Based on the CVD theory, liquid drops of SiBN are initially formed on the substrate which solidify to form the SiBN film. One droplet grows to a bubble of cauliflower-like structure. The chemical reaction rate in CVD process increases with the concentration of reactants. With the increase of SiCl_4_ flow rate, increased sizes of SiBN droplets are formed on the substrate, creating bigger cauliflower-like structure. When the droplets are big enough, they will spread and contact to each other, forming a flat structure and then causing the decrease of cauliflower-like structure. When the chemical reaction reaches a certain level, small spherical particles are nucleated in gas phase, which adhere on the surface of the sample by physical or chemical adsorption. This phenomenon, so-called gas-phase nucleation [15], can result in great fall of deposition rate for it consumes many reactants. To explain the influence of SiCl_4_ flow rate on gas-phase nucleation, the equations below are needed.

(4) $$r_{c}=\frac{{2\sigma V}_{m}}{RT\gamma }$$

(5) $$J=\left({4\pi r}_{c}^{2}\right)\cdot \alpha_{c}{P(2\pi mRT)}^{-\frac{1}{2}}\cdot n_{i}\cdot \exp(-\frac{\Delta G_{c}}{RT})$$

where *r_c_* is the critical nucleation radius; *σ* is surface energy per unit area; *V_m_* is the molecule volume of species at nucleation temperature; *R* is the mole gas constant (*R* = 8.314 J/(mol K)); *γ* is the degree of super saturation; *J* is the nuclei formation rate; *α_c_* is the condensation coefficient of nuclei; *P* is the vapor pressure of reactant; *n_i_* is the concentration of nuclei; *α_c_P*(2π*mRT*)^−1/2^ is the kinetic frequency factor; and −Δ*G_c_* is the critical Gibbs energy of nuclei formation. According to Equations (4) and (5), the gas-phase nuclei formation rate increases with the rise of the vapor pressure of reactant which is proportional to the SiCl_4_ flow. Therefore, more nuclei are formed in the gas environment, creating small spherical particles.

The cross-sectional morphologies at various SiCl_4_ flow rates are showed in Figure 3. It is concluded that SiCl_4_ flow rate has a significant effect on the thickness of SiBN coating. When SiCl_4_ flow rate is 10 mL/min, the thickness of the SiBN coating on the surface of the carbon cloth (outer) is 10 μm and the thickness of the coating surrounds (inner) the fibers is 1 μm, which accounts for the comparatively low deposition rate. With the increase of SiCl_4_ flow rate, the deposition rate increases, leading to more SiBN deposited on the surface of the cloth and less deposited inside it. When the SiCl_4_ flow rate is 20 mL/min, the deposition thickness on the cloth is 50 μm and inside the cloth is 200 nm. When the flow rate arrives 30 mL/min, the deposition thickness on the surface is 40 μm and inside the cloth is about 200 nm.

The deposition rate increases with the increase of SiCl_4_ flow rate; thus, more SiBN ceramic will be deposited on the surface of carbon cloth. As the gas-phase nucleation occurs, the thickness of SiBN coating on the surface decreases due to the consumption of reactants in the gas environment. On the contrary, increased deposition rate leads to thinner SiBN coating deposited inside the carbon cloth. The thickness gradient of SiBN coating inside and outside increases as well. Infiltration depth and uniformity mainly depend on the deposition rate and the diffusion in carbon cloth, and the deposition rate plays a more important role at high temperature. The effective diffusion coefficient (*D_eff_*) is based on the Fick diffusion coefficient (*D_F_*) and the Knudsen diffusion coefficient (*D_K_*) with the Equation (6).

(6) $$\frac{1}{D_{\mathrm{eff}}}=\frac{1}{D_{F}}+\frac{1}{D_{K}}$$

At the beginning of deposition, the pore diameter is big enough to allow the reactant molecules diffuse into the carbon cloth by Fick diffusion. The diffusion is mainly related to temperature and pressure as Equation (7) shows.

(7) $$D_{F}=D_{0}T^{m}P^{-1}$$

where *D_0_* refers to a constant; *T* refers to temperature (K); *P* refers to pressure (Pa); 1.5 < *m* < 2. During this time, the deposition inside the cloth is mainly determined by the reaction. As more material is deposited on the surface, the pores which allow gas molecules to enter the cloth become smaller. When the pore diameter is comparable to or smaller than the mean free path of the diffusing reactant molecules, the Knudsen diffusion occurs.

(8) $$D_{K}=\frac{d_{P}}{3}{\left(\frac{8RT}{M}\right)}^{3/2}$$

where *d_P_* refers to the pore diameter; *T* refers to the temperature; *M* refers to the amount of reactants; *R* refers to mole gas constant (*R* = 8.314 J/(mol K)). Equation (8) shows that Knudsen diffusion is relevant to the type of reactant, temperature, and pore diameter, but has nothing to do with the pressure which is a big difference from Fick diffusion. The gas molecules collide with the pore walls more frequently than with each other, so that it is hard to diffuse. During this time, the deposition inside the cloth is mainly determined by the diffusion. The more SiCl_4_ is consumed in the deposition, the quicker the deposition reaches the second stage. It is the reason that the thickness of SiBN coating inside the carbon cloth deposited at 20 mL/min is much thinner than that at 10 mL/min. With the continuous increase of SiCl_4_ flow rate, the thickness on the edge levels off. For the internal pores within carbon cloth, thickness of coatings on the internal fibers is independent with the reactant content and is related to temperature and the pore size, according to Equation (7). Thus, before the closure of the pore channel on the edge occurred, the diffusion and deposition on the internal fibers almost did not change when SiCl_4_ flow rate increased from 20 mL/min to 30 mL/min.

### 3.3. Phase Composition, Chemical Composition, and Bonding States

**Figure 4:**
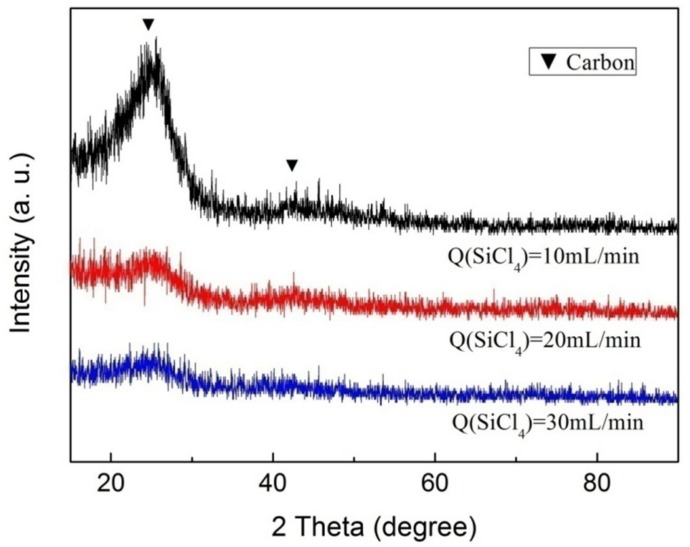
X-ray powder diffraction (XRD) patterns of SiBN ceramic deposited at various SiCl_4_ flow rates.

The composition and crystallinity of the SiBN coating deposited at various SiCl_4_ flow rates are examined by X-ray powder diffraction (XRD), as shown in Figure 4. There are no BN and Si_3_N_4_ diffraction peaks, and the strong graphite peaks at 26.603° and 44.666° come from the substrate of carbon cloth. It indicates that the deposition products are X-ray amorphous, which is consistent with the result analyzed with transmission electron microscopy (TEM) in our previous work [14]. The peaks of graphite at 10 mL/min are much stronger than those at 20 mL/min and 30 mL/min, especially the peak at 26.603°. It is because that the thickness of SiBN coating grows with the increase of SiCl_4_ flow rate.

To determine the element types, element contents, and chemical bonding states of the SiBN coating, XPS analysis is carried out. Table 2 shows the elements and their contents in SiBN coating deposited at various SiCl_4_ flow rates. As can be seen, Si, B, N, and O are observed in all the products. B content decreases with the increase of SiCl_4_ flow rate, while Si content increases and O content remains relatively steady in the vicinity of 14–17 at.%.

XPS is such a kind of surface analysis technology whose test depth is generally ranged from a few to tens of nanometers. In order to protect the bonding on the surface from being damaged, the samples are not etched before the test. The active part, lowly crystalline BN, on the surface is easy to be oxidized while being exposed in the air. It is the reason why the content of O element is a bit high.

**Figure 5:**
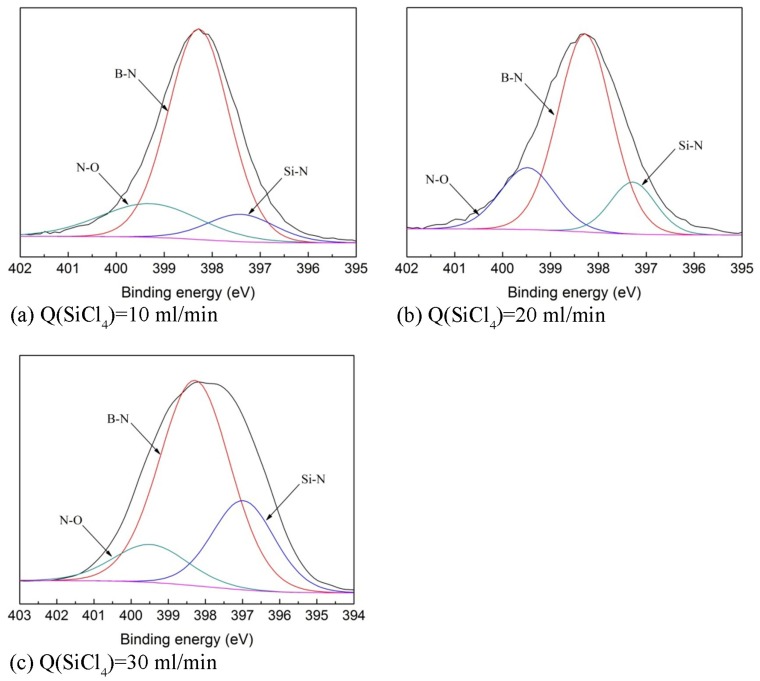
N1s spectra of SiBN ceramic deposited at various SiCl_4_ flow rates (**a**) Q(SiCl_4_) = 10 mL/min; (**b**) Q(SiCl_4_) = 20 mL/min; (**c**) Q(SiCl_4_) = 30 mL/min.

Characterization of the chemical bonding in the SiBN coating is done with XPS, which offers the composition and the bonding types of atoms. The XPS data is transformed into N1s spectra according to the relationship between the Si, B, N, and O element bonding states and bonding energy, as shown in Figure 5. It shows that the bonding types are the same at various SiCl_4_ flow rates. There are three main types of bonding in SiBN coating—namely Si-N, B-N, and N-O—which are consistent with our previous work about the effect of deposition temperature on the deposition [13]. However, the content of them varies at various SiCl_4_ flow rates, as shown in Table 3. With the increase of SiCl_4_ flow rate, the content of B-N bonding decreases, while that of Si-N bonding increases. As for N-O bonding, there is not obvious rule about it. This content goes up and down at various SiCl_4_ flow rates. This is because N-O bonding is formed due to the oxidation of the surface when samples are exposed in the air as discussed above.

### 3.4. Deposition Mechanism

The deposition rate, chemical composition, and bonding states are all determined by the deposition chemical reactions of the gas system. In the gas system of SiCl_4_-NH_3_-BCl_3_-H_2_-Ar, the main reaction equations are:

(9) BCl_3_(g) + NH_3_(g) = BN(s) + 3 HCl(g)

(10) 3/4 SiCl_4_(g) + NH_3_(g) = 1/4 Si_3_N_4_(s) + 3 HCl(g)

At 900 °C, reaction (9) is the dominant reaction because the Gibbs energy (∆G) of Equation (9), −191.414 KJ (calculated with Factsage software, Thermofact /CRCT and GTT-Technologies, Montreal, Canada and Herzogenrath, Germany) is much lower than that of reaction (10), −91.768 KJ. Therefore, the content of B element is much higher than Si and the main bonding state of N element is B-N, as shown in Table 2 and Table 3. The deposition of SiBN coating can be regarded as the co-deposition of X-ray amorphous BN and Si_3_N_4_ at 900 °C. During the deposition, reaction (9) and reaction (10) are simultaneous, forming the B-N and Si-N. Different SiCl_4_ flow rates lead to different ratios of BN and Si_3_N_4_. As the SiCl_4_ flow rate increases from 10 mL/min to 20 mL/min, reaction (10) is promoted whereas reaction (9) is influenced slightly, when NH_3_ is in excess compared with SiCl_4_ and BCl_3_. As a consequence, the deposition rate largely improves. This results in the increase of Si element in the coating and thus in the observed increase of Si-N bonding. However, when the SiCl_4_ flow rate increases from 20 mL/min to 30 mL/min, reaction (10) is promoted greatly, and there is not enough NH_3_ to be consumed in reaction (9). As a result, the amount of B is reduced as shown by the reduction of the B-N bonding XPS peak. On the contrary, Si content increased as shown by the increase of the Si-N bonding XPS peak. At 900 °C, reaction (9) is still the main reaction. Because reaction (10) is promoted and (9) is restrained, the deposition rate is reduced at this range of the SiCl_4_ flow rate.

## 4. Conclusions

The influence of the SiCl_4_ flow rate on the deposition rate, morphology, phase composition, chemical composition, and bonding states of SiBN coating deposited by low pressure chemical vapor deposition (LPCVD) at 900 °C has been investigated using SiCl_4_-BCl_3_-NH_3_-H_2_-Ar gas environment in this study. The deposition rate increases at first and then decreases with the increase of SiCl_4_ flow rate. The surface of the coating is a uniform cauliflower-like structure at 10 mL/min and 20 mL/min, and covered with small spherical particles at 30 mL/min. The coating deposited at various SiCl_4_ flow rates is X-ray amorphous, and contains Si, B, N, and O elements. The main bonding states are B-N, Si-N, and N-O. The amount of B element and B-N bonding decrease with the increase of SiCl_4_ flow rate, while Si element and Si-N bonding increase. The main deposition mechanism refers to two parallel reactions of BCl_3_+NH_3_ and SiCl_4_+NH_3_. The deposition process is mainly controlled by the reaction of BCl_3_+NH_3_.

## Figures and Tables

**Table 1:** Deposition parameters of SiBN coating with SiCl_4_-BCl_3_-NH_3_-H_2_-Ar system via low pressure chemical vapor deposition (LPCVD)

Substrate	T (°C)	T (h)	Q_SiCl4_	Q_BCl3_	Q_NH3_	Q_H2_ (Dilution)	Q_Ar_	P (kPa)
			(mL/min)	(mL/min)	(mL/min)	(mL/min)	(mL/min)	
Carbon cloth	900	7	102030	20	60	100	100	1

**Table 2:** Element content of SiBN ceramic deposited at various SiCl_4_ flow rates analyzed by X-ray photoelectron spectroscopy (XPS).

SiCl_4_ Flow Rate (mL/min)	Element Content (at.%)			
	B	N	Si	O
10	42.10	36.88	6.33	14.69
20	31.09	38.73	14.30	15.88
30	30.38	33.82	18.81	16.98

**Table 3:** Bonding states of N1s and bonding content of SiBN coatings deposited at various SiCl_4_ flow rates.

SiCl_4_ Flow Rate (mL/min)	Bonding Content (at.%)		
	N-B (398.28 eV)	N-Si (397.40 eV)	N-O (399.28 eV)
10	71.11	10.27	18.62
20	65.01	14.34	20.65
30	63.45	24.25	12.30
